# Glue Ear, Hearing Loss and IQ: An Association Moderated by the Child’s Home Environment

**DOI:** 10.1371/journal.pone.0087021

**Published:** 2014-02-03

**Authors:** Amanda J. Hall, Richard Maw, Elizabeth Midgley, Jean Golding, Colin Steer

**Affiliations:** 1 Children’s Hearing Centre, University Hospitals Bristol NHS Foundation Trust, Bristol, United Kingdom; 2 Centre for Hearing and Balance Studies, University of Bristol, Bristol, United Kingdom; 3 Centre for Child and Adolescent Health, University of Bristol, Bristol, United Kingdom; Alberta Provincial Laboratory for Public Health/University of Alberta, Canada

## Abstract

**Background:**

Glue ear or otitis media with effusion (OME) is common in children and may be associated with hearing loss (HL). For most children it has no long lasting effects on cognitive development but it is unclear whether there are subgroups at higher risk of sequelae.

**Objectives:**

To examine the association between a score comprising the number of times a child had OME and HL (OME/HL score) in the first four/five years of life and IQ at age 4 and 8. To examine whether any association between OME/HL and IQ is moderated by socioeconomic, child or family factors.

**Methods:**

Prospective, longitudinal cohort study: the Avon Longitudinal Study of Parents and Children (ALSPAC). 1155 children tested using tympanometry on up to nine occasions and hearing for speech (word recognition) on up to three occasions between age 8 months and 5 years. An OME/HL score was created and associations with IQ at ages 4 and 8 were examined. Potential moderators included a measure of the child’s cognitive stimulation at home (HOME score).

**Results:**

For the whole sample at age 4 the group with the highest 10% OME/HL scores had performance IQ 5 points lower [95% CI −9, −1] and verbal IQ 6 points lower [95% CI −10, −3] than the unaffected group. By age 8 the evidence for group differences was weak. There were significant interactions between OME/HL and the HOME score: those with high OME/HL scores and low 18 month HOME scores had lower IQ at age 4 and 8 than those with high OME/HL scores and high HOME scores. Adjusted mean differences ranged from 5 to 8 IQ points at age 4 and 8.

**Conclusions:**

The cognitive development of children from homes with lower levels of cognitive stimulation is susceptible to the effects of glue ear and hearing loss.

## Introduction

Glue ear or otitis media with effusion (OME) is one of the most common conditions of childhood. It is generally accepted that for most children, transient OME has only a minimal effect on development, such as speech and language outcomes [1]. However the risk to development may be greater for children with OME which persists over time and any developmental impact is hypothesised to occur only in those cases with a resultant hearing loss, particularly if it occurs during sensitive periods of development [2]–[3]. Research into developmental outcomes of OME should therefore account not only for persistence of OME but also for any associated hearing loss.

Additionally if OME and hearing loss is coupled with other risks such as intellectual disabilities or existing sensorineural hearing loss the developmental impact may be greater. For this reason taking a cumulative risk approach to study the association between OME, hearing loss and development is a generally accepted strategy [3]; OME and hearing loss are not examined in isolation but in the context of the child’s exposure to other risks to delayed development (moderators), which may include socioeconomic factors, parental and child characteristics [4]–[5].

Several prospective studies of OME, hearing loss and cognition have shown associations between measures of early OME and hearing history and IQ [6]–[8] although other studies found no association [9]–[12]. These studies covered a range of sizes and populations and not all examined hearing levels or the influence of moderators.

The aim of this study was to prospectively examine the association between episodes of OME and hearing loss over the first 4 to 5 years of life and IQ at ages 4 and 8 years in the Avon Longitudinal Study of Parents and Children (ALSPAC). We tested the hypothesis that child and environmental factors moderate associations between OME, hearing loss and IQ. We took prospective, serial measures of OME and hearing loss to account for its known fluctuating nature. Longitudinal measures of hearing enabled us to differentiate between OME related hearing loss and sensorineural hearing loss. We prospectively measured characteristics of the child and family which could act as moderators or confounders.

## Methods

### Ethics Statement

Ethical approval for the study was obtained from the ALSPAC Ethics and Law Committee and the Local Research Ethics Committees. The ethics committee specifically approved the questionnaires and the clinic testing protocols including the methods of gaining consent.

For the self-completion questionnaire data, consent was implied when postal questionnaires were returned. ALSPAC is a longitudinal study with many contact points with participants, therefore all questionnaires to participants were logged when sent, reminded and returned, as were requests not to send further questionnaires.

For the clinic data used in this study, verbal consent was obtained from the parents or guardians on behalf of the children and verbal assent from the children was always obtained before all measures. Verbal consent was used as many measures were taken at each half day clinic and many of these were repeat measures from earlier clinics. It was ensured that all participants were quite clear what was involved with each measure and that they could withdraw at any time. It was considered burdensome to ask participants to supply written consent for every measure. All written consent forms and data sheets are filed securely and logged electronically.

### Participants

ALSPAC recruited 14,541 pregnant women resident in Avon, UK with expected dates of delivery between 1st April 1991 and the 31st December 1992 [13]. This study comprised the Children in Focus (CiF) group, a 10% sample of the ALSPAC cohort who attended clinics at the University of Bristol at various time intervals between 4 to 61 months of age. The CiF group were chosen at random from the last 6 months of ALSPAC births (1432 families attended at least one clinic). Excluded were those mothers who had moved out of the area or were lost to follow-up, and those enrolled in another study of infant development in Avon.

For this study children were excluded if English was not spoken at home (as the outcome measures were English language based), if the child attended fewer than 4 tympanometry assessments or if the child had a sensorineural hearing loss.

### Measurement of OME and Hearing Loss

The CiF clinics of relevance to this study were the nine measures of middle ear function using tympanometry up to age 5 (at 8, 12, 18, 25, 31, 37, 43, 49 and 61 months) [14] and measures of word recognition threshold using a test of the binaural ability to hear speech, at 2 ½, 3 ½ and 5 years [15].

Tympanometry was used to determine the presence of middle ear effusion at each visit. Tympanograms were coded according to Jerger’s modified Fieullau-Nikolajsen method [16]. We used the approach taken by Wilson [17] to score these coded tympanograms at each visit, see Table S1 for detail. Each ear of each child was scored according to whether it was free of OME (type A or C1 tympanogram: 0 points), had negative middle ear pressure (type C2: ½ point) or had evidence of OME (type B: 1 point). The score for each child at each visit was summed to give a total OME score.

Binaural hearing ability was assessed using the Automated McCormick Toy Test to measure the word recognition threshold (WRT), which is strongly related to hearing ability [18]. The WRT was measured at 2 ½, 3 ½ and 5 years using only toys the names of which the child recognised to ensure as much as possible that the test reflected hearing ability rather than language. The standard clinical cut-off value of 35 dB was taken to differentiate between normal and abnormal hearing (>20 dB HL) in the better hearing ear [18]. Based on the measured WRT at each occasion, children were given the following scores: < = 35 dB scored 0, 36–45 dB scored 1, >45 dB WRT 2.

Exclusion of those with sensorineural hearing loss was based on the results of longitudinal pure tone audiometry available up to age 11 [19]. Cases where tympanometry and audiograms (air and bone conduction) indicated unilateral or bilateral sensorineural hearing loss (greater than 20 dB averaged over 0.5–4 kHz in either ear) were excluded from the study.

### Defining the OME and Hearing Loss Exposure

We took the approach to combine the OME and hearing loss scores over the first five years to generate a single, cumulative exposure score, named the *OME/HL score*. This approach was taken firstly in view of the hypothesised importance of persistent on-going exposure over transient, episodic exposure [3]. The second reason was due to limitations of the ALSPAC hearing data: unlike other studies of OME, hearing loss and development (e.g. [12]) in which hearing was assessed at the same time as tympanometry and so the impact of OME and hearing loss could be examined as independent exposures, in ALSPAC measures of hearing loss were only available on three out of the nine occasions at which tympanometry was measured. No hearing measurements were taken before age 2 ½ years and the maximum number of hearing tests available for analysis of the 4 year IQ measure was two; a third measure was available at age 5. As a consequence, the available data were likely to underestimate the effect of hearing loss making it difficult to separate out the contributions of OME and HL. In view of these limitations we combined the OME and hearing loss scores. The aim was to use all available data on the premise that hearing loss provided additional information on the severity of a child’s exposure than OME alone.

For outcomes assessed at 4 years, the OME/HL score was based on tests up to and including age 4 years. A maximum number of eight sessions could give a maximum possible score of 20 (eight bilateral B tympanograms and two WRT>45 dB). For outcomes assessed at 8 years, the OME/HL score was based on tests up to and including 5 years. A maximum number of nine sessions could give a maximum possible score of 24 (nine bilateral B tympanograms and three WRT>45 dB).

Using a combined score enabled us to deal with the issue of missing data using a simple prorating method. Such a method would not have been straightforward with separate OME and hearing loss measures. While prorating may indicate a total score for the missing measurements, it does not solve the issue of how this total should be apportioned between multiple time points. A prorated score was calculated for those children who did not attend the maximum number of sessions by converting their score to be out of either 20 or 24 (for example, a child who attended four sessions and had three bilateral B tympanograms would have their score of 6 out of a maximum possible score of 8 prorated to 18).

The OME/HL score therefore encapsulates three groups of children: those with no OME or hearing loss, those with OME and no hearing loss and those with OME and hearing loss.

### Measurement of IQ

At age 4, IQ was measured using the Wechsler Pre-school and Primary Scale of Intelligence, WPPSI [20]. At age 8 years, IQ was measured using the Wechsler Intelligence Scale for Children, WISC-III [21]. At this age a shortened version was used with alternate items applied for all subtests except the coding subtest in which all the items were applied. Testing was carried out by trained psychologists and measures of verbal and performance IQ were obtained. The testers were blind to the OME and hearing history of the child.

### Confounders and Moderators

A range of confounders was adjusted for in the analyses. These were socioeconomic factors: highest maternal education level achieved (categorised in 3 groups – *low*/minimal or vocational qualifications, *medium*/qualifications obtained at age 16 e.g. O’Levels and *high*/qualifications obtained at age 18 years or above e.g. A’Levels or degree); housing tenure (categorised as mortgaged/owned, private rented, council/housing association/other) and parental social class (categorised as manual, non-manual based on highest occupation level of mother or father). Also maternal and child factors: maternal age; parity (categorised as 0, 1–2, ≥3); smoking in 1^st^ three months of pregnancy (yes/no); smoking in last two weeks of pregnancy (yes/no); birthweight; gestational age (<37 weeks/37 weeks or higher) and sex of child.

Information on the child’s cognitive environment at home was derived from maternal questionnaires based on the HOME inventory [22]. Questions included whether the child owns cuddly toys, push/pull toys, coordination toys, number of books, whether the mother attempts to teach the child and whether the mother talks to the child. This HOME score was calculated at 6, 18, 30 and 42 months.

A parenting score measuring parenting style was derived from maternal questionnaires asking whether the mother plays with the child, sings to the child, shows or reads books to the child, cuddles the child, takes the child for walks, or similar depending on the age of the child. The score was calculated when the child was 6, 18, 24, 38 and 42 months.

All confounders were considered as potential moderators.

### Statistical Analysis

The exposure OME/HL score was investigated as a categorical variable to explore potential non linear effects. Three categories were derived: a group unaffected by OME or hearing loss (OME/HL score of 0), a group with the highest 10% scores (most affected) and a group of those cases remaining (intermediate group) with mild/moderate OME or hearing loss (the remaining cases).

Regression analysis was used to examine differences in IQ according to OME/HL group. Unadjusted analyses and analyses adjusted for confounders are presented. The OME/HL score was also investigated as a continuous variable.

Nonlinear effects were tested for by comparing the results using OME/HL as a categorical variable with those using the OME/HL score as a continuous variable. The difference in explanation between these two models can be used as a test for the deviation from linearity.

Statistical interactions to identify possible susceptible subgroups were tested in two stages. Firstly in a two variable model with the OME/HL variable and the moderator variable. Secondly those moderator variables for which there was strong evidence of an interaction were included within the fully adjusted model to explore whether the interactions were an artefact of confounding. The p for interaction reflected a hierarchical model such that interactions were ‘adjusted’ not only for confounders but also for OME/HL and moderator main effects.

All analyses were conducted using STATA version 11.0.

## Results

### Sample

**Figure 1:**
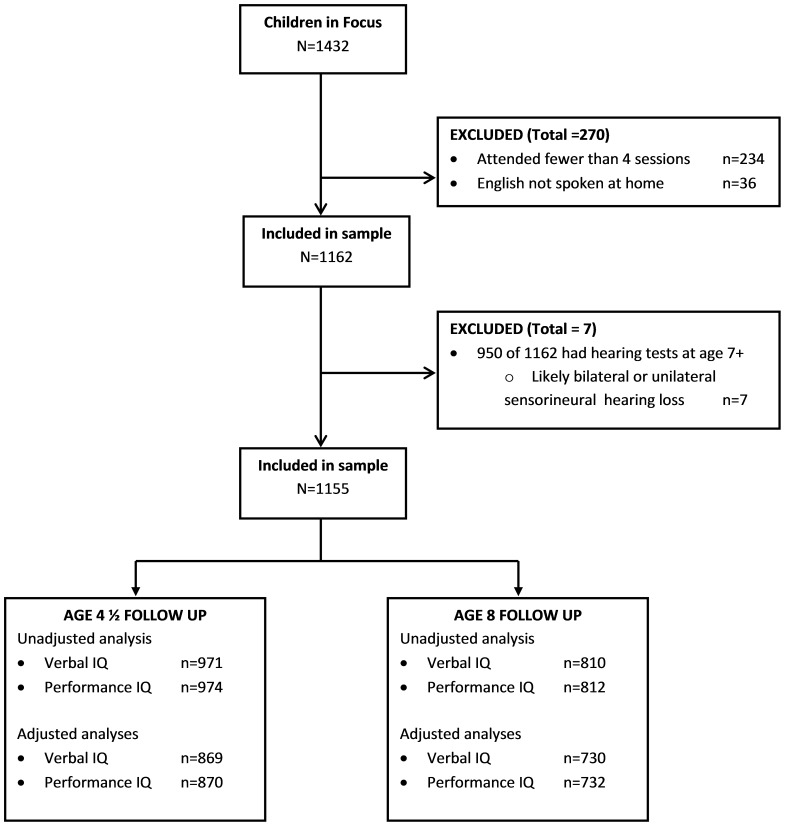
Participant flow diagram. Variables included in adjusted analyses were maternal education level, housing tenure, parental social class, maternal age, parity, smoking during 1st three months of pregnancy, smoking in last two weeks of pregnancy, birthweight, gestational age, sex of child.

The original CIF group included 1432 children. Following the exclusion criteria, the sample size was reduced to 1155 children; see the flow chart of participants in Figure 1. Characteristics of the study sample are shown in Table 1.

**Table 1:** Characteristics of the study sample (N = 1155).

	n (%)
**Highest maternal education level**	
Low	262 (22.68)
Medium	404 (34.98)
High	457 (39.57)
Missing	32 (2.77)
**Maternal age**	
<20	24 (2.08)
20–24	137 (11.86)
25–29	472 (40.86)
30–34	391 (33.86)
35+	131 (11.34)
Missing	0
**Housing tenure**	
Owned/mortgaged	919 (79.57)
Private rented	63 (5.45)
Council/other	151 (13.08)
Missing	22 (1.90)
**Parental social class**	
Manual	164 (14.20)
Non-manual	888 (76.88)
Missing	103 (8.92)
**Sex of child**	
Male	625 (54.11)
Female	530 (45. 89)
Missing	0
**Ethnicity**	
White	1085 (93.94)
Non-white	26 (2.25)
Missing	44 (3.81)
**Mean birthweight**, g (SD)	3446.02 (521.5)
**Mean gestation**, weeks (SD)	39.51 (1.624)

### OME/HL Scores and Groups

By age 4, 47% had complete tympanometry data and the average missingness was 1 out of 8 sessions. By age 5, 43% had complete tympanometry data and the average missingness was 1.25 sessions out of 9. A summary of the tympanometry and WRT scores measured at each time point is shown in Table S2.

**Figure 2:**
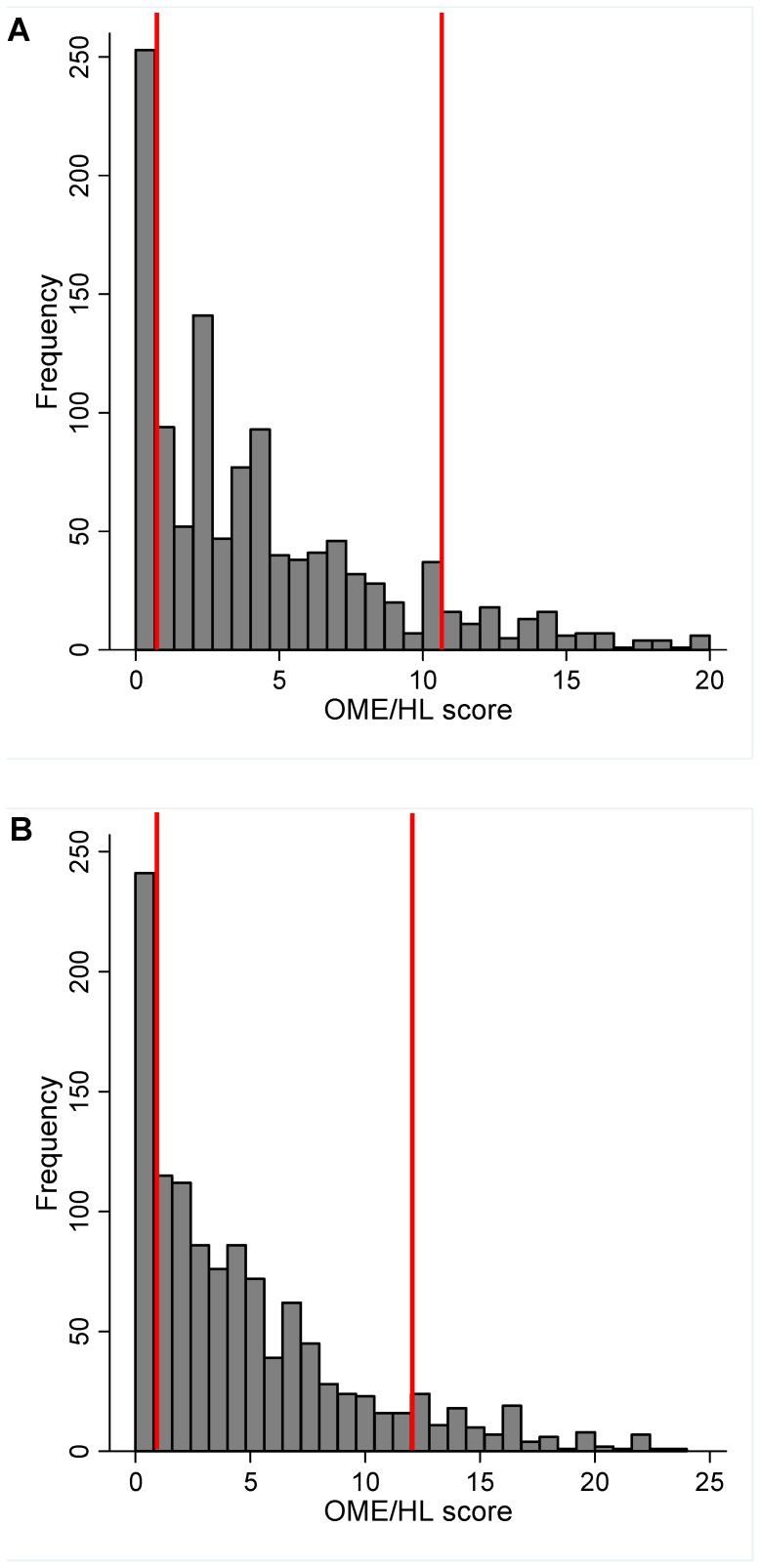
Distribution of the prorated OME/HL score and the OME/HL groups. A) At four years (maximum score 20). B) At five years (maximum score 24). The cut-off points defining the unaffected (score of zero), intermediate and highest 10% groups are shown.

The prorated OME/HL scores at four and five years are shown in Figure 2. This shows a left skewed distribution of OME/HL score with most children having a low or zero score over the first four and five years of life. The group with the highest 10% of scores was reflected as those with OME/HL scores of 11 to 20 at age four and 12 to 24 at age five. The unaffected group was defined as those with scores of zero and the intermediate group was defined as the remaining cases.

### Associations between OME/HL and Cognition

The association between OME/HL group and IQ was examined (see Table S3 for a summary of the IQ scores at age 4 and 8). At age 4 there was evidence of a strong association between OME/HL group and both verbal and performance IQ. Tables 2 and 3 compare the IQ of the intermediate and highest 10% OME/HL groups to the unaffected group. For those in the highest 10% group, the adjusted verbal IQ was 6 points lower than the unaffected group and performance IQ was 5 points lower. For the intermediate group there was a difference of 2 points in verbal IQ. Adjustment for confounders made little difference to the effect sizes.

**Table 2:** Differences in mean verbal IQ score at age 4 according to OME/HL group (up to 4 years).

	Unadjusted model^a^			Adjusted model^b^			Adjusted model^c^		
OME/HL group	Coefficient [95% CI]	N	P-value^d^	Coefficient [95% CI]	N	P-value^d^	Coefficient [95% CI]	N	P-value^d^
Unaffected	Reference	188		Reference	163		Reference	140	
Intermediate	−2.51 [−4.64, −0.38]	684	0.021	−2.43 [−4.53, −0.33]	618	0.023	−2.48 [−4.70, −0.26]	516	0.028
Highest 10% scores	−7.38 [−10.59, −4.17]	99	≤0.001	−7.17 [−10.30, −4.03]	88	≤0.001	−6.75 [−10.10, −3.39]	73	≤0.001
**P for trend** e	<0.001			<0.001			<0.001		
**P for deviation** **from linearity**	0.220			0.219			0.377		

a A negative coefficient indicates that IQ is lower in the OME/HL group compared to the unaffected reference group.

b Adjusted for maternal education level, housing tenure, parental social class, maternal age, parity, smoking during 1^st^ 3 months of pregnancy, smoking last 2 weeks of pregnancy, birthweight, gestational age, sex of child.

c Adjusted for maternal education level, housing tenure, parental social class, maternal age, parity, smoking during 1^st^ 3 months of pregnancy, smoking last 2 weeks of pregnancy, birthweight, gestational age, sex of child, HOME and parenting scores.

d p-value for testing the effect of each OME/HL group vs the reference unaffected group.

e p-value for linear trend in effect size across groups.

**Table 3:** Differences in mean performance IQ score at age 4 according to OME/HL group (up to 4 years).

	Unadjusted model^a^			Adjusted model^b^			Adjusted model^c^		
OME/HL group	Coefficient [95% CI]	N	P-value^d^	Coefficient [95% CI]	N	P-value^d^	Coefficient [95% CI]	N	P-value^d^
Unaffected	Reference	189		Reference	164		Reference	141	
Intermediate	−0.63 [−2.95, 1.69]	686	0.595	−0.12 [−2.49, 2.24]	618	0.917	−0.86 [−3.34, 1.60]	516	0.491
Highest10% scores	−5.9 [−9.41, −2.39]	99	0.001	−5.16 [−8.70, −1.62]	88	0.004	−5.09 [−8.84, −1.35]	73	0.008
**P for trend** e	0.001			0.004			0.02		
**P for deviation from linearity**	0.027			0.020			0.135		

a A negative coefficient indicates that IQ is lower in the OME/HL group compared to the unaffected reference group.

b Adjusted for maternal education level, housing tenure, parental social class, maternal age, parity, smoking during 1^st^ 3 months of pregnancy, smoking last 2 weeks of pregnancy, birthweight, gestational age, sex of child.

c Adjusted for maternal education level, housing tenure, parental social class, maternal age, parity, smoking during 1^st^ 3 months of pregnancy, smoking last 2 weeks of pregnancy, birthweight, gestational age, sex of child, home and parenting scores.

d p-value for testing the effect of each OME/HL group vs the unaffected reference group.

e p-value for linear trend in effect size across groups.

At age 8, there was a reduction of approximately 4 points in verbal IQ and of 3 points in performance IQ in children with the highest OME/HL scores (Tables 4 and 5). These relationships showed weak or no statistical significance in the reduced sample available at 8 years.

**Table 4:** Differences in mean verbal IQ score at age 8 according to OME/HL group (up to 5 years).

	Unadjusted model^a^			Adjusted model^b^			Adjusted model^c^		
OME/HL group	Coefficient [95% CI]	N	P-value^d^	Coefficient [95% CI]	N	P-value^d^	Coefficient [95% CI]	N	P-value^d^
Unaffected	Reference	142		Reference	127		Reference	112	
Intermediate	−0.27 [−3.29, 2.73]	584	0.858	0.08 [−2.93, 3.10]	527	0.955	−0.18 [−3.37, 3.00]	452	0.910
Highest 10%scores	−3.98 [−8.42, 0.44]	84	0.078	−3.91 [−8.33, 0.50]	76	0.082	−4.79 [−9.51, −0.08]	65	0.046
**P for trend** e	0.136			0.106			0.0721		
**P for deviation from linearity**	0.192			0.120			0.116		

a A negative coefficient indicates that IQ is lower in the OME/HL group compared to the unaffected reference group.

b Adjusted for maternal education level, housing tenure, parental social class, maternal age, parity, smoking during 1^st^ 3 months of pregnancy, smoking last 2 weeks of pregnancy, birthweight, gestational age, sex of child.

c Adjusted for maternal education level, housing tenure, parental social class, maternal age, parity, smoking during 1^st^ 3 months of pregnancy, smoking last 2 weeks of pregnancy, birthweight, gestational age, sex of child, home and parenting scores.

d p-value for testing the effect of each OME/HL group vs the unaffected reference group.

e p-value for linear trend in effect size across groups.

**Table 5:** Differences in mean performance IQ score at age 8 according to OME/HL group (up to 5 years).

	Unadjusted model^a^			Adjusted model^b^			Adjusted model^c^		
OME/HL group	Coefficient [95% CI]	N	P-value^d^	Coefficient [95% CI]	N	P-value^d^	Coefficient [95% CI]	N	P-value^d^
Unaffected	Reference	141		Reference	126		Reference	111	
Intermediate	0.04 [−3.03, 3.11]	586	0.979	0.33 [−2.89, 3.55]	529	0.840	0.87 [−2.47, 4.22]	454	0.608
Highest 10%scores	−3.95 [−8.45, 0.55]	85	0.085	−3.77 [−8.46, 0.91]	77	0.115	−3.25 [−8.17, 1.66]	66	0.194
**P for trend** e	0.1154			0.126			0.151		
**P for deviation from linearity**	0.132			0.112			0.089		

a A negative coefficient indicates that IQ is lower in the OME/HL group compared to the unaffected reference group.

b Adjusted for maternal education level, housing tenure, parental social class, maternal age, parity, smoking during 1^st^ 3 months of pregnancy, smoking last 2 weeks of pregnancy, birthweight, gestational age, sex of child.

c Adjusted for maternal education level, housing tenure, parental social class, maternal age, parity, smoking during 1^st^ 3 months of pregnancy, smoking last 2 weeks of pregnancy, birthweight, gestational age, sex of child, home and parenting scores.

d p-value for testing the effect of each OME/HL group vs the unaffected reference group.

e p-value for linear trend in effect size across groups.

There was some minor evidence of non-linearity at age 8, although overall the linear analyses were more appropriate. The interaction terms were omitted from these models which may have contributed to these minor non-linear effects.

### Interactions

Possible interactions between the OME/HL variables and a range of moderators were tested (see Table S4 for a descriptive summary of the HOME scores and Tables S5–10 for the full results of the interaction analysis). This showed that HOME scores at age 6, 18 and 42 months were significant moderators of the effect of OME/HL score on verbal IQ at age 4. HOME scores at 6, 18, 30 and 42 months were significant moderators on performance IQ at age 4.

HOME scores at 18 and 30 months and parenting score at 38 months were significant moderators of the effect of OME/HL score on verbal IQ at age 8. HOME scores at 18 and 30 months and parenting at 6 months were significant moderators on performance IQ.

**Figure 3:**
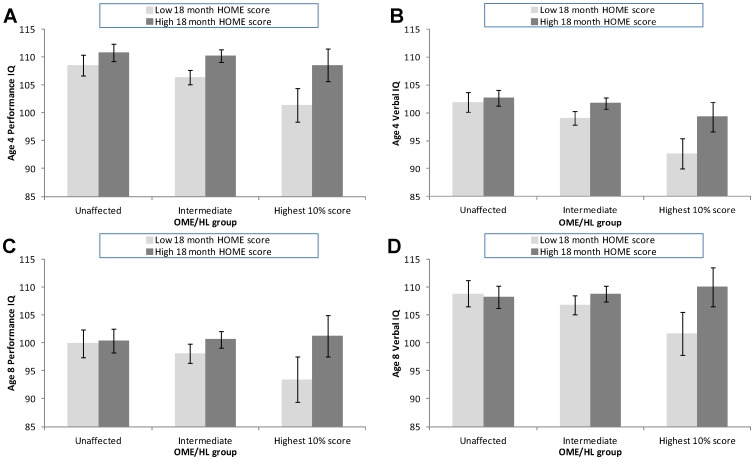
Adjusted mean IQ (95% CI) according to OME/HL group and 18 month HOME scores. A) Performance IQ at 4 years. B) Verbal IQ at 4 years. C) Performance IQ at 8 years. D) Verbal IQ at 8 years. Bottom 50% HOME scores: light grey bars; top 50% HOME scores: dark grey bars (HOME score groupings are for illustration only; statistical analyses use the raw HOME scores). Mean scores adjusted for maternal education level, housing tenure, parental social class, maternal age, parity, smoking during 1st three months of pregnancy, smoking last two weeks of pregnancy, birthweight, gestational age, sex of child. There was evidence of an interaction between OME/HL and HOME scores such that those children with poor scores on both measures performed much worse than other groups (p for interaction in adjusted model using linear scores: p = 0.008 (A), p = 0.001 (B), p = 0.006 (C), p = 0.008 (D)).

Figure 3 shows the adjusted mean IQ at age 4 and 8 according to OME/HL group and 18 month HOME score (see Figures S1 and S2 for HOME score interactions at other ages). At age 4, there was a general trend for IQ to decrease as the severity of the OME/HL group increased; the trend was more marked for those with lower HOME scores. At age 8, the trend of decreasing IQ with increasing severity of OME/HL group was only observed for those with low but not high HOME scores. In fact there is an observed slight but non-significant trend where those with high HOME scores and high OME/HL scores had higher mean IQ than those with an OME/HL score of zero but this trend was likely to be due to chance (verbal IQ p = 0.265; performance IQ p = 0.685).

### Linear Effects of OME/HL

Analyses were also performed treating the OME/HL score as a dimensional measure (see Tables S5–8 & 11, 12). These analyses did not materially add to the main effects noted in the categorical results but provided stronger evidence for the interaction effects.

### Missing Data

We examined the sensitivity of results to prorating as a method of imputation for cases with missing tympanometry data. The data were analysed by restricting to those cases with a complete set of tympanometry values. We compared effect sizes from these analyses to the results using the full data set which included both complete and prorated values. This showed minimal differences in effect sizes between the two analyses.

### Validity of Composite score

The validity of calculating a composite OME and HL score from the individual tympanometry and hearing variables was examined. The main effect analyses were performed using the individual tympanometry and hearing loss scores as exposure variables in place of the composite score. The hypothesis was tested that the contribution of the individual OME and HL effects are equal at each time point; the results of the F test are consistent with this hypothesis (at age 4½ verbal IQ p = 0.119; performance IQ p = 0.444) and support the use of a composite score.

Analyses were also conducted in which the OME/HL composite score was separated into an OME composite score and separate hearing loss variables; see Table S13 and S14 for results at age 4. This indicated that both OME and hearing loss at age 2 ½ were significantly related to verbal IQ and that hearing loss at age 2 ½ was related to performance IQ; however interpretation was limited by the lack of hearing data at the earlier ages.

We examined the results of unadjusted interaction analyses using OME and HL as separate variables focusing on the 18 month HOME scores as the moderator variable and IQ at age 8 as the outcome variable. There was a significant interaction between the OME score and the HOME score (p = 0.016), and between hearing loss at age 2 ½ year and the HOME score (p = 0.010) on performance IQ. There were no significant interactions with the HOME score and hearing loss at 3 ½ (p = 0.244) or 5 years (p = 0.546). For verbal IQ, both OME and hearing loss at age 2 ½ showed significant interactions with the HOME score (p = 0.001 and 0.029 respectively) but not hearing loss at age 3 ½ and 5 (p = 0.106 and 0.149 respectively). As described for the main effects analyses, it is not clear if the results observed for the OME score alone would persist if hearing loss data were available for each time point below the age of 2 ½ years.

## Discussion

This study examined the cognitive development of a group of children with high OME/HL scores over the first 4 to 5 years of their life and compared their development to children with no recorded episodes of OME or hearing loss during this time. The group with the highest 10% scores had lower verbal and performance IQ by approximately 5 to 6 points compared to those unaffected by OME or hearing loss at age 4; this effect was diminished by age 8.

We examined whether there were factors that moderated this association and showed that the home environment had a consistent moderating effect on OME/HL score; such that children with high OME/HL *and* low HOME scores had lower verbal and performance IQ than children with high OME/HL but high HOME scores. This difference was observed at both age 4 and 8 and ranged from 5 to 8 IQ points.

Our results at a population level are consistent with other prospective cohort studies showing an association, albeit small, between OM, hearing history and early life IQ which diminishes with age. The 1970 British Birth Cohort Study showed associations between reported hearing loss in the first 5 years with verbal and performance IQ at age 5 but not 10 [6]. The Dunedin study showed associations with verbal but not performance IQ at age 11 [7]; the Greater Boston OM Study showed associations at age 7 with verbal and performance IQ [8] and Johnson et al [23] found an association at age 3 but not at 5 or 7. Those that showed no association were the smaller study by Gravel and Wallace [24] at age 4 and the detailed study by Roberts et al [9]–[12] examining the cognitive outcomes of a small group of African American children, who found no association between ages 2 and 12.

Of those studies examining the moderating effect of the child’s home environment on the relationship between OME, hearing loss and cognition, Johnson et al [23] showed a moderating effect of both the HOME score and socioeconomic status on the relationship between OM group and IQ at age 3, but no interactions were observed at later ages. Roberts et al [11]–[12] examined the interaction between OM, hearing history and the HOME scores and found no evidence of a moderating effect of the home environment on OME or hearing loss. Both these studies had smaller sample sizes than the current study and may not have had the statistical power to detect interactions. Interestingly Roberts et al did find that children with OME or hearing loss were more likely to live in less responsive home environments; we did not observe this in our study, and differences in IQ were still present even after adjustment for HOME and parenting scores.

The current study identified a group of children within the population, those from home environments with lower levels of cognitive stimulation, who are more vulnerable to the effects of OME and hearing loss. The interaction between OME, hearing loss and the home environment on cognitive development is likely to be complex. It is well established that children from homes with limited cognitive stimulation have poorer cognitive development in early and later childhood [25]–[27]. The presence of a hearing loss may compound limited cognitive stimulation at home by further reducing access to verbal interactions and incidental learning. We observed associations with both verbal and performance IQ indicating that the effect is unlikely to be solely mediated by language. There is evidence that the increased listening effort for children with hearing loss compared to those with normal hearing reduces the cognitive resources available for other non-auditory tasks [28]. If cognitive resources are reduced as well as cognitive stimulation, these effects may multiply. Due to the plasticity of the auditory system, on resolution of OME related hearing loss the negative effects are usually compensated for [29], as was seen for the whole sample at age 8. However when accompanied by another risk factor the results of this study show the impact may be longer lasting.

The strengths of this study are the prospective nature of the design and the number of cases allowing statistical interactions to be examined. The focus on the worst 10% of cases, those persistently affected, gives the study direct clinical applicability unlike studies which do not differentiate between episodic versus continuous OME. However the main limitation was that unlike the studies by Roberts et al [11]–[12], our study did not have concurrent hearing and tympanometry data available at all time points, which did not enable us to separate either the main and interaction effects as owing to OME or to hearing loss. The measures of hearing were only available on three occasions, at age 2 ½ and later, so the cumulative measure does not take into account hearing ability in the first two years of life. Analyses which examined the OME and hearing loss variables separately indicated that hearing loss contributes to both the main and interaction effects, in keeping with developmental models of OME [2]–[3]. The effects were strongest with earlier rather than later hearing loss consistent with a possible sensitive period [2]. An effect was also observed for OME but interpretation of this is hampered by the lack of hearing data at the other time points; it is not clear whether this is a real effect of OME or an artefact of missing hearing data.

The study sample was shifted towards the more advantaged end of the population [13], which may have biased the results. We did not find any strong evidence for socioeconomic confounding and adjustment for socioeconomic variables did not markedly reduce effect sizes. Hence we would expect than the impact of any differential drop-out would be minimal. In any observational study, there is the possibility of residual confounding and although we controlled for a wide range of confounders this remains a possibility.

This research has important clinical implications. Management of OME in the UK has altered significantly over the past few years with a tendency not to treat for long periods of time. This refers to the generality of the condition. Clinicians and commissioners must be aware that there are susceptible subgroups that could be more disadvantaged by such a reluctance to treat. The group identified in this study may be just one of several. This study highlights the importance of taking a cumulative risk approach to management of ‘glue ear’ and clinicians, parents, commissioners and policy makers should be alert to subgroups. Future research should focus on how to identify in clinical practice susceptible children at risk and develop interventions targeting modifiable factors, particularly the home learning environment and the hearing of the child, which may protect against these effects.

## Supporting Information

Figure S1 **Adjusted mean IQ (95% CI) at age 4 years according to OME/HL group and HOME scores.** A) Performance IQ, HOME score 6 months. B) Verbal IQ, HOME score 6 months. C) Performance IQ, HOME score 18 months. D) Verbal IQ, HOME score 18 months. E) Performance IQ, HOME score 30 months. F) Verbal IQ, HOME score 30 months. G) Performance IQ, HOME score 42 months. H) Verbal IQ, HOME score 42 months. Bottom 50% HOME scores: light grey bars; top 50% HOME scores: dark grey bars (HOME score groupings are for illustration only; statistical analyses use the raw HOME scores). Mean scores adjusted for maternal education level, housing tenure, parental social class, maternal age, parity, smoking during 1st 3 months of pregnancy, smoking last 2 weeks of pregnancy, birthweight, gestational age, sex of child. There was evidence of an interaction between OME/HL and HOME scores such that those children with poor scores on both measures performed much worse than other groups (p for interaction in adjusted model using linear scores: p = 0.050 (A), p = 0.005 (B), p = 0.008 (C), p≤0.001 (D), p = 0.005 (E), p = 0.022 (F), p = 0.031 (G), p = 0.002 (H)).
(TIF)

Figure S2 **Adjusted mean IQ (95% CI) at age 8 years according to OME/HL group and HOME scores.** A) Performance IQ, HOME score 6 months. B) Verbal IQ, HOME score 6 months. C) Performance IQ, HOME score 18 months. D) Verbal IQ, HOME score 18 months. E) Performance IQ, HOME score 30 months. F) Verbal IQ, HOME score 30 months. G) Performance IQ, HOME score 42 months. H) Verbal IQ, HOME score 42 months. Bottom 50% HOME scores: light grey bars; top 50% HOME scores: dark grey bars (HOME score groupings are for illustration only; statistical analyses use the raw HOME scores). Mean scores adjusted for maternal education level, housing tenure, parental social class, maternal age, parity, smoking during 1st 3 months of pregnancy, smoking last 2 weeks of pregnancy, birthweight, gestational age, sex of child. There was evidence of an interaction between OME/HL and HOME scores such that those children with poor scores on both measures performed much worse than other groups (p for interaction in adjusted model using linear scores: p = 0.118 (A), p = 0.178 (B), p = 0.006 (C), p = 0.008 (D), p = 0.013 (E), p = 0.025 (F), p = 0.093 (G), p = 0.116 (H)).
(TIF)

Table S1 Scoring of tympanograms. Key: A or C1 normal middle ear function/mild negative middle ear pressure; C2 negative middle ear pressure; B indicates middle ear effusion; G grommet; P perforation.
(DOCX)

Table S2 Number (percentage) cases according to tympanometry and word recognition scores.
(DOCX)

Table S3 Descriptive statistics for the IQ outcome measures.
(DOCX)

Table S4 Descriptive statistics for the HOME measures.
(DOCX)

Table S5 Interactions between moderators and OME/HL score (continuous variable) on verbal IQ at age 4 years. ^a^ Adjusted for maternal education level, housing tenure, parental social class, maternal age, parity, smoking during 1st 3 months of pregnancy, smoking last 2 weeks of pregnancy, birthweight, gestational age, sex of child, HOME and parenting scores**.** ^b^ Moderators included if there was evidence of a significant interaction**.** ^c^ Coefficient of OME/HL and moderator interaction. The interaction effects reflect the change in the OME/HL effect compared to the reference level for parity or for a one unit change in the HOME score. Since the OME/HL effect is negative, positive interactions reflect an ameliorating effect.
(DOCX)

Table S6 Interactions between moderators and OME/HL score (continuous variable) on performance IQ at age 4 years. ^a^ Adjusted for maternal education level, housing tenure, parental social class, maternal age, parity, smoking during 1st 3 months of pregnancy, smoking last 2 weeks of pregnancy, birthweight, gestational age, sex of child, HOME and parenting scores. ^b^ Moderators included if there was evidence of a significant interaction. ^c^ Coefficient of OME/HL and moderator interaction. The interaction effects reflect the change in the OME/HL for a one unit change in the HOME or parenting score. Since the OME/HL effect is negative, positive interactions reflect an ameliorating effect.
(DOCX)

Table S7 Interactions between moderators and OME/HL score (continuous variable) on verbal IQ at age 8 years. ^a^ Adjusted for maternal education level, housing tenure, parental social class, maternal age, parity, smoking during 1st 3 months of pregnancy, smoking last 2 weeks of pregnancy, birthweight, gestational age, sex of child, HOME and parenting scores. ^b^ Moderators included if there was evidence of a significant interaction. ^c^ Coefficient of OME/HL and moderator interaction. The interaction effects reflect the change in the OME/HL effect for a one unit change in the HOME or parenting score. Since the OME/HL effect is negative, positive interactions reflect an ameliorating effect.
(DOCX)

Table S8 Interactions between moderators and OME/HL score (continuous variable) on performance IQ at age 8 years. ^a^ Adjusted for maternal education level, housing tenure, parental social class, maternal age, parity, smoking during 1st 3 months of pregnancy, smoking last 2 weeks of pregnancy, birthweight, gestational age, sex of child, HOME and parenting scores. ^b^ Moderators included if there was evidence of a significant interaction. ^c^ Coefficient of OME/HL and moderator interaction. The interaction effects reflect the change in the OME/HL effect compared to the reference level for not smoking in pregnancy (no smoking) or for a one unit change in the HOME or parenting score. Since the OME/HL effect is negative, positive interactions reflect an ameliorating effect.
(DOCX)

Table S9 Interactions between moderators and OME/HL (categorical variable) on verbal IQ at age 8 years. ^a^Adjusted for maternal education level, housing tenure, parental social class, maternal age, parity, smoking during 1st 3 months of pregnancy, smoking last 2 weeks of pregnancy, birthweight, gestational age, child sex, home and parenting scores. ^b^ Moderators included if there was evidence of a significant interaction. ^c^ Coefficient of OME/HL and moderator interaction. The interaction effects reflect the change in the OME/HL effect for a one unit change in the HOME score. Since the OME/HL effect is negative, positive interactions reflect an ameliorating effect.
(DOCX)

Table S10 Interactions between moderators and OME/HL (categorical variable) on performance IQ at age 8 years. ^a^ Adjusted for maternal education level, housing tenure, parental social class, maternal age, parity, smoking during 1st 3 months of pregnancy, smoking last 2 weeks of pregnancy, birthweight, gestational age, sex of child, HOME and parenting scores. ^b^ Moderators included if there was evidence of a significant interaction. ^c^ Coefficient of OME/HL and moderator interaction. The interaction effects reflect the change in the OME/HL effect compared to the reference level for not smoking in pregnancy (no smoking) or for a one unit change in the HOME score. Since the OME/HL effect is negative, positive interactions reflect an ameliorating effect.
(DOCX)

Table S11 Association between OME/HL score (continuous) and verbal IQ at age 4 and 8. ^a^ A negative coefficient indicates that as the OME/HL severity score increases, IQ decreases. ^b^ Adjusted for maternal education level, housing tenure, parental social class, maternal age, parity, smoking during 1st 3 months of pregnancy, smoking last 2 weeks of pregnancy, birthweight, gestational age, sex of child. ^c^ Adjusted for maternal education level, housing tenure, parental social class, maternal age, parity, smoking during 1st 3 months of pregnancy, smoking last 2 weeks of pregnancy, birthweight, gestational age, sex of child, HOME and parenting scores.
(DOCX)

Table S12 Association between OME/HL score (continuous) and performance IQ at age 4 and 8. ^a^ A negative coefficient indicates that as the OME/HL severity score increases, IQ decreases. ^b^ Adjusted for maternal education level, housing tenure, parental social class, maternal age, parity, smoking during 1st 3 months of pregnancy, smoking last 2 weeks of pregnancy, birthweight, gestational age, sex of child. ^c^ Adjusted for maternal education level, housing tenure, parental social class, maternal age, parity, smoking during 1st 3 months of pregnancy, smoking last 2 weeks of pregnancy, birthweight, gestational age, sex of child, HOME and parenting scores.
(DOCX)

Table S13 Differences in mean verbal IQ score at age 4 according to separate OME group and hearing loss variables (up to 4 years). ^a^ Hearing loss categorised as WRT >35 dBA. ^b^ Fully adjusted for all confounders and HOME/parenting scores.
(DOCX)

Table S14 Differences in mean performance IQ score at age 4 according to separate OME group and hearing loss variables (up to 4 years). ^a^ Hearing loss categorised as WRT >35 dBA. ^b^ Fully adjusted for all confounders and HOME/parenting scores.
(DOCX)
