# Supplementary figures and images for: Glue Ear, Hearing Loss and IQ: An Association Moderated by the Child’s Home Environment
Source: PLoS One. 2014 Feb 3;9(2):e87021. doi: 10.1371/journal.pone.0087021 (PMC3911938; doi:10.1371/journal.pone.0087021)

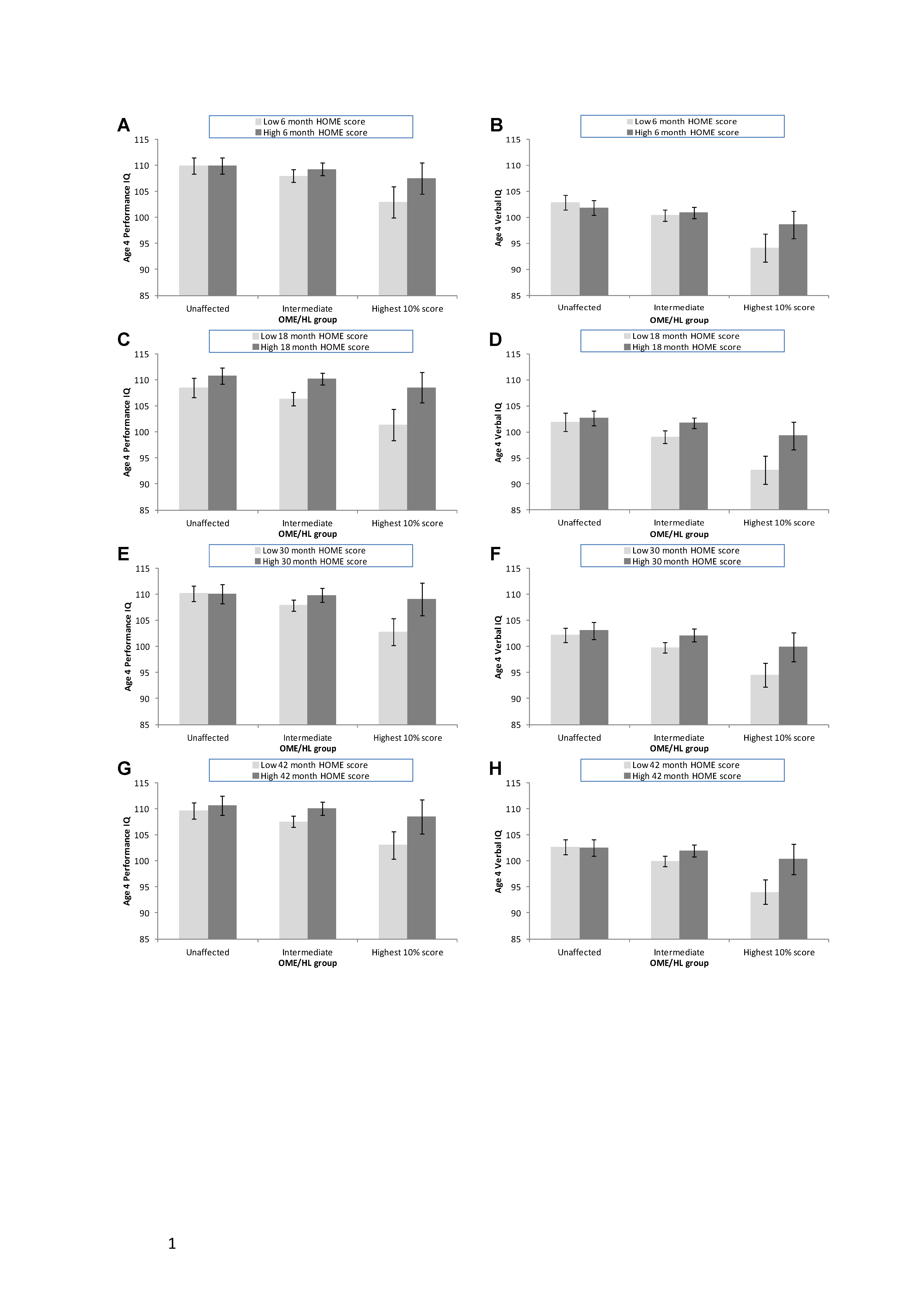

Supplement: Figure S1 — Adjusted mean IQ (95% CI) at age 4 years according to OME/HL group and HOME scores. A) Performance IQ, HOME score 6 months. B) Verbal IQ, HOME score 6 months. C) Performance IQ, HOME score 18 months. D) Verbal IQ, HOME score 18 months. E) Performance IQ, HOME score 30 months. F) Verbal IQ, HOME score 30 months. G) Performance IQ, HOME score 42 months. H) Verbal IQ, HOME score 42 months. Bottom 50% HOME scores: light grey bars; top 50% HOME scores: dark grey bars (HOME score groupings are for illustration only; statistical analyses use the raw HOME scores). Mean scores adjusted for maternal education level, housing tenure, parental social class, maternal age, parity, smoking during 1st 3 months of pregnancy, smoking last 2 weeks of pregnancy, birthweight, gestational age, sex of child. There was evidence of an interaction between OME/HL and HOME scores such that those children with poor scores on both measures performed much worse than other groups (p for interaction in adjusted model using linear scores: p = 0.050 (A), p = 0.005 (B), p = 0.008 (C), p≤0.001 (D), p = 0.005 (E), p = 0.022 (F), p = 0.031 (G), p = 0.002 (H)). (TIF) [file pone.0087021.s001.tif]

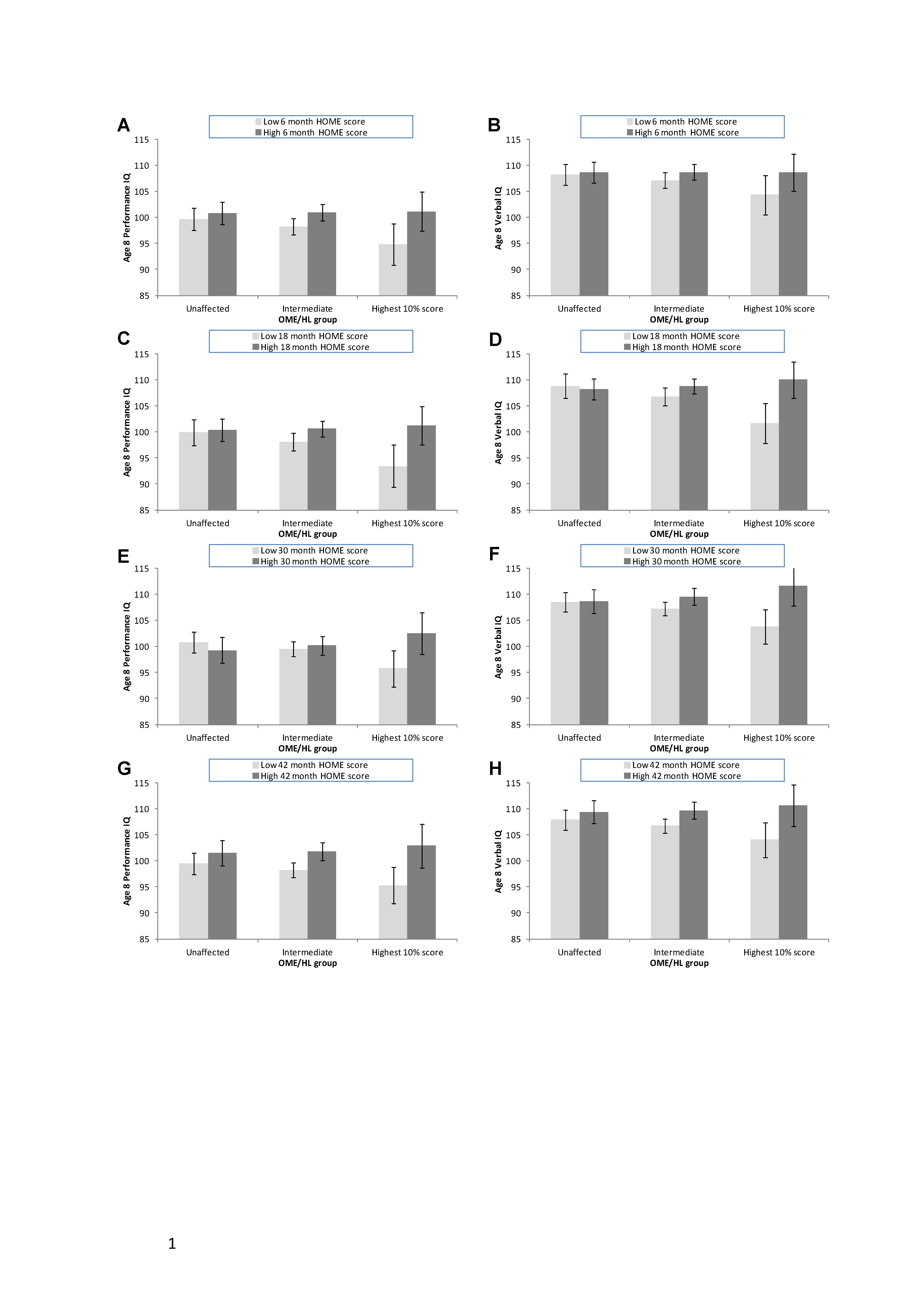

Supplement: Figure S2 — Adjusted mean IQ (95% CI) at age 8 years according to OME/HL group and HOME scores. A) Performance IQ, HOME score 6 months. B) Verbal IQ, HOME score 6 months. C) Performance IQ, HOME score 18 months. D) Verbal IQ, HOME score 18 months. E) Performance IQ, HOME score 30 months. F) Verbal IQ, HOME score 30 months. G) Performance IQ, HOME score 42 months. H) Verbal IQ, HOME score 42 months. Bottom 50% HOME scores: light grey bars; top 50% HOME scores: dark grey bars (HOME score groupings are for illustration only; statistical analyses use the raw HOME scores). Mean scores adjusted for maternal education level, housing tenure, parental social class, maternal age, parity, smoking during 1st 3 months of pregnancy, smoking last 2 weeks of pregnancy, birthweight, gestational age, sex of child. There was evidence of an interaction between OME/HL and HOME scores such that those children with poor scores on both measures performed much worse than other groups (p for interaction in adjusted model using linear scores: p = 0.118 (A), p = 0.178 (B), p = 0.006 (C), p = 0.008 (D), p = 0.013 (E), p = 0.025 (F), p = 0.093 (G), p = 0.116 (H)). (TIF) [file pone.0087021.s002.tif]
