# Supplementary material for: Glue Ear, Hearing Loss and IQ: An Association Moderated by the Child’s Home Environment
Source: PLoS One. 2014 Feb 3;9(2):e87021. doi: 10.1371/journal.pone.0087021 (PMC3911938; doi:10.1371/journal.pone.0087021)
Supplement: Table S1 — Scoring of tympanograms. Key: A or C1 normal middle ear function/mild negative middle ear pressure; C2 negative middle ear pressure; B indicates middle ear effusion; G grommet; P perforation. (DOCX) [file pone.0087021.s003.docx]

|  |  | Right ear | | |
| --- | --- | --- | --- | --- |
|  | Tympanogram type | A or C1 (=0) | C2 (=0.5) | B or G or P (=1) |
| Left ear | A or C1 (=0) | 0 | 0.5 | 1 |
|  | C2 (=0.5) | 0.5 | 1 | 1.5 |
|  | B or G or P (=1) | 1 | 1.5 | 2 |
