# Supplementary material for: Glue Ear, Hearing Loss and IQ: An Association Moderated by the Child’s Home Environment
Source: PLoS One. 2014 Feb 3;9(2):e87021. doi: 10.1371/journal.pone.0087021 (PMC3911938; doi:10.1371/journal.pone.0087021)
Supplement: Table S2 — Number (percentage) cases according to tympanometry and word recognition scores. (DOCX) [file pone.0087021.s004.docx]

| Age (months) | Tympanometry scores | | | | | | Word recognition scores | | | |
| --- | --- | --- | --- | --- | --- | --- | --- | --- | --- | --- |
|  | 0 | 0.5 | 1 | 1.5 | 2 | Missing | 0 | 1 | 2 | Missing |
| 8 | 564 (48.83) | 27 (2.34) | 188 (16.28) | 18 (1.56) | 256 (22.16) | 102 (8.83) |  |  |  |  |
| 12 | 624 (54.03) | 42 (3.64) | 179 (15.50) | 23 (1.99) | 145 (12.55) | 142 (12.29) |  |  |  |  |
| 18 | 536 (46.41) | 54 (4.68) | 148 (12.81 ) | 41 (3.55) | 231 (20.0 ) | 145 (12.55) |  |  |  |  |
| 25 | 729 (63.12) | 34 (2.94) | 104 (9.00) | 22 (1.90) | 92 (7.97) | 174 (15.06) |  |  |  |  |
| 31 | 691 (59.83) | 43 (3.72) | 114 (9.87) | 27 (2.34) | 142 (12.29) | 138 (11.95) | 723 (62.60) | 138 (11.95 ) | 68 (5.89) | 226 (19.57) |
| 37 | 773 (66.93) | 30 (2.60) | 80 (6.93) | 13 (1.13) | 94 (8.14) | 165 (14.29) |  |  |  |  |
| 43 | 645 55.84) | 69 (5.97) | 125 (10.82) | 39 (3.38) | 123 (10.65) | 154 (13.33) | 901 ( 78.01) | 68 (5.89) | 27 (2.34) | 159 (13.77) |
| 49 | 724 (62.68) | 49 (4.24) | 89 (7.71) | 27 (2.34) | 68 (5.89) | 198 (17.14) |  |  |  |  |
| 61 | 676 (58.53) | 53 (4.59) | 85 (7.36) | 27 (2.34) | 77 (6.67) | 237 (20.52) | 869 (75.24) | 39 (3.38) | 17 (1.47) | 230 (19.91) |
