# Supplementary material for: Glue Ear, Hearing Loss and IQ: An Association Moderated by the Child’s Home Environment
Source: PLoS One. 2014 Feb 3;9(2):e87021. doi: 10.1371/journal.pone.0087021 (PMC3911938; doi:10.1371/journal.pone.0087021)
Supplement: Table S3 — Descriptive statistics for the IQ outcome measures. (DOCX) [file pone.0087021.s005.docx]

| Age (years) |  | Mean | SD | Range | n |
| --- | --- | --- | --- | --- | --- |
| 4 | Performance IQ | 108.03 | 14.49 | 55-151 | 974 |
|  | Verbal IQ | 100.28 | 13.30 | 54-152 | 971 |
| 8 | Performance IQ | 99.16 | 16.73 | 46-140 | 812 |
|  | Verbal IQ | 107.61 | 16.43 | 52-153 | 810 |
