# Supplementary material for: Glue Ear, Hearing Loss and IQ: An Association Moderated by the Child’s Home Environment
Source: PLoS One. 2014 Feb 3;9(2):e87021. doi: 10.1371/journal.pone.0087021 (PMC3911938; doi:10.1371/journal.pone.0087021)
Supplement: Table S4 — Descriptive statistics for the HOME measures. (DOCX) [file pone.0087021.s006.docx]

| Age (years) | 25^th^ percentile | Median | 75^th^ percentile | Range | n |
| --- | --- | --- | --- | --- | --- |
| 6 months | 7 | 8 | 10 | 2-12 | 1115 |
| 18 months | 9 | 11 | 12 | 5-12 | 1103 |
| 30 months | 11 | 12 | 13 | 4-13 | 1074 |
| 42 months | 12 | 13 | 14 | 5-15 | 1047 |
