# Supplementary material for: Glue Ear, Hearing Loss and IQ: An Association Moderated by the Child’s Home Environment
Source: PLoS One. 2014 Feb 3;9(2):e87021. doi: 10.1371/journal.pone.0087021 (PMC3911938; doi:10.1371/journal.pone.0087021)
Supplement: Table S5 — Interactions between moderators and OME/HL score (continuous variable) on verbal IQ at age 4 years. a Adjusted for maternal education level, housing tenure, parental social class, maternal age, parity, smoking during 1st 3 months of pregnancy, smoking last 2 weeks of pregnancy, birthweight, gestational age, sex of child, HOME and parenting scores. b Moderators included if there was evidence of a significant interaction. c Coefficient of OME/HL and moderator interaction. The interaction effects reflect the change in the OME/HL effect compared to the reference level for parity or for a one unit change in the HOME score. Since the OME/HL effect is negative, positive interactions reflect an ameliorating effect. (DOCX) [file pone.0087021.s007.docx]

|  | | **Unadjusted model** | | | **Fully adjusted model^a^** | | |
| --- | --- | --- | --- | --- | --- | --- | --- |
| **Moderator variable^b^** | | **Interaction coefficient [95% CI]^c^** | **P-value** | **N** | **Interaction coefficient [95% CI]^c^** | **P-value** | **N** |
| HOME score | 6 months | 0.14[0.04, 0.24] | 0.006 | 946 | 0.14 [0.04, 0.25] | 0.005 | 729 |
|  | 18 months | 0.20 [0.07, 0.32] | 0.002 | 940 | 0.27 [0.12, 0.41] | <0.001 | 729 |
|  | 42 months | 0.13 [0.00, 0.25] | 0.044 | 914 | 0.21 [0.07, 0.35] | 0.002 | 729 |
| Parity | 0 | Reference | Reference | 950 | Reference | Reference | 729 |
|  | 1-2 | -0.37 [-0.77, 0.03] | 0.071 |  | -0.17 [-0.59, 0.25] | 0.430 |  |
|  | ≥3 | -0.94 [-1.79, -0.09] | 0.029 |  | -0.63 [-1.64, 0.38] | 0.222 |  |
