# Supplementary material for: Glue Ear, Hearing Loss and IQ: An Association Moderated by the Child’s Home Environment
Source: PLoS One. 2014 Feb 3;9(2):e87021. doi: 10.1371/journal.pone.0087021 (PMC3911938; doi:10.1371/journal.pone.0087021)
Supplement: Table S6 — Interactions between moderators and OME/HL score (continuous variable) on performance IQ at age 4 years. a Adjusted for maternal education level, housing tenure, parental social class, maternal age, parity, smoking during 1st 3 months of pregnancy, smoking last 2 weeks of pregnancy, birthweight, gestational age, sex of child, HOME and parenting scores. b Moderators included if there was evidence of a significant interaction. c Coefficient of OME/HL and moderator interaction. The interaction effects reflect the change in the OME/HL for a one unit change in the HOME or parenting score. Since the OME/HL effect is negative, positive interactions reflect an ameliorating effect. (DOCX) [file pone.0087021.s008.docx]

|  | | **Unadjusted model** | | | **Fully adjusted model^a^** | | |
| --- | --- | --- | --- | --- | --- | --- | --- |
| **Moderator variable^b^** | | **Interaction coefficient [95% CI]^c^** | **P-value** | **N** | **Interaction coefficient [95% CI]^c^** | **P-value** | **N** |
| HOME score | 6 months | 0.15 [0.04, 0.26] | 0.005 | 949 | 0.11 [0.00, 0.23] | 0.050 | 730 |
|  | 18 months | 0.20 [0.06, 0.34] | 0.004 | 943 | 0.21 [0.05, 0.37] | 0.008 | 730 |
|  | 30 months | 0.20 [0.05, 0.34] | 0.006 | 922 | 0.24 [0.07, 0.41] | 0.005 | 730 |
|  | 42 months | 0.14 [0.00, 0.28] | 0.037 | 917 | 0.16 [0.01, 0.32] | 0.031 | 730 |
| Parenting | 18 months | 0.04 [0.00, 0.09] | 0.044 | 943 | 0.04 [0.00, 0.09] | 0.099 | 730 |
|  | 24 months | 0.08 [0.00, 0.17] | 0.047 | 882 | 0.06 [-0.02, 0.15] | 0.179 | 730 |
|  | 38 months | 0.09 [0.01, 0.16] | 0.018 | 919 | 0.08 [0.00, 0.16] | 0.058 | 730 |
