# Supplementary material for: Glue Ear, Hearing Loss and IQ: An Association Moderated by the Child’s Home Environment
Source: PLoS One. 2014 Feb 3;9(2):e87021. doi: 10.1371/journal.pone.0087021 (PMC3911938; doi:10.1371/journal.pone.0087021)
Supplement: Table S7 — Interactions between moderators and OME/HL score (continuous variable) on verbal IQ at age 8 years. a Adjusted for maternal education level, housing tenure, parental social class, maternal age, parity, smoking during 1st 3 months of pregnancy, smoking last 2 weeks of pregnancy, birthweight, gestational age, sex of child, HOME and parenting scores. b Moderators included if there was evidence of a significant interaction. c Coefficient of OME/HL and moderator interaction. The interaction effects reflect the change in the OME/HL effect for a one unit change in the HOME or parenting score. Since the OME/HL effect is negative, positive interactions reflect an ameliorating effect. (DOCX) [file pone.0087021.s009.docx]

|  | | **Unadjusted model** | | | **Fully adjusted model^a^** | | |
| --- | --- | --- | --- | --- | --- | --- | --- |
| **Moderator variable^b^** | | **Interaction coefficient [95% CI]^c^** | **P-value** | **N** | **Interaction coefficient [95% CI]^c^** | **P-value** | **N** |
| HOME score | 6 months | 0.16 [0.03, 0.28] | 0.013 | 798 | 0.09 [-0.04, 0.22] | 0.178 | 629 |
|  | 18 months | 0.28 [0.12, 0.43] | 0.001 | 789 | 0.24 [0.06, 0.42] | 0.008 | 629 |
|  | 30 months | 0.21 [0.06, 0.37] | 0.006 | 779 | 0.21 [0.02, 0.41] | 0.025 | 629 |
| Parenting score | 6 months | 0.16 [0.01, 0.31] | 0.031 | 797 | 0.13 [-0.02, 0.29] | 0.107 | 629 |
|  | 38 months | 0.10 [0.02, 0.18] | 0.013 | 771 | 0.10 [0.01, 0.19] | 0.025 | 629 |
|  | 42 months | 0.06 [0.00, 0.11] | 0.026 | 772 | 0.00 [-0.04, 0.06] | 0.754 | 629 |
