# Supplementary material for: Glue Ear, Hearing Loss and IQ: An Association Moderated by the Child’s Home Environment
Source: PLoS One. 2014 Feb 3;9(2):e87021. doi: 10.1371/journal.pone.0087021 (PMC3911938; doi:10.1371/journal.pone.0087021)
Supplement: Table S8 — Interactions between moderators and OME/HL score (continuous variable) on performance IQ at age 8 years. a Adjusted for maternal education level, housing tenure, parental social class, maternal age, parity, smoking during 1st 3 months of pregnancy, smoking last 2 weeks of pregnancy, birthweight, gestational age, sex of child, HOME and parenting scores. b Moderators included if there was evidence of a significant interaction. c Coefficient of OME/HL and moderator interaction. The interaction effects reflect the change in the OME/HL effect compared to the reference level for not smoking in pregnancy (no smoking) or for a one unit change in the HOME or parenting score. Since the OME/HL effect is negative, positive interactions reflect an ameliorating effect. (DOCX) [file pone.0087021.s010.docx]

|  | | **Unadjusted model** | | | **Fully adjusted model^a^** | | |
| --- | --- | --- | --- | --- | --- | --- | --- |
| **Moderator variable^b^** | | **Interaction coefficient [95% CI]^c^** | **P-value** | **N** | **Interaction coefficient [95% CI]^c^** | **P-value** | **N** |
| HOME score | 6 months | 0.14 [0.01, 0.27] | 0.026 | 800 | 0.11 [-0.02, 0.24] | 0.118 | 631 |
|  | 18 months | 0.21 [0.04, 0.37] | 0.011 | 791 | 0.26 [0.07, 0.44] | 0.006 | 631 |
|  | 30 months | 0.22 [0.06, 0.38] | 0.006 | 781 | 0.25 [0.05, 0.45] | 0.013 | 631 |
| Parenting score | 6 months | 0.16 [0.00, 0.31] | 0.042 | 799 | 0.19 [0.02, 0.36] | 0.024 | 631 |
| Smoking in pregnancy | Last 2 weeks | -0.82 [-1.48, -0.16] | 0.014 | 803 | -0.79 [-1.59, 0.00] | 0.052 | 631 |
