# Supplementary material for: Glue Ear, Hearing Loss and IQ: An Association Moderated by the Child’s Home Environment
Source: PLoS One. 2014 Feb 3;9(2):e87021. doi: 10.1371/journal.pone.0087021 (PMC3911938; doi:10.1371/journal.pone.0087021)
Supplement: Table S9 — Interactions between moderators and OME/HL (categorical variable) on verbal IQ at age 8 years. aAdjusted for maternal education level, housing tenure, parental social class, maternal age, parity, smoking during 1st 3 months of pregnancy, smoking last 2 weeks of pregnancy, birthweight, gestational age, child sex, home and parenting scores. b Moderators included if there was evidence of a significant interaction. c Coefficient of OME/HL and moderator interaction. The interaction effects reflect the change in the OME/HL effect for a one unit change in the HOME score. Since the OME/HL effect is negative, positive interactions reflect an ameliorating effect. (DOCX) [file pone.0087021.s011.docx]

|  |  | **Unadjusted model** | | | **Fully adjusted model^a^** | | |
| --- | --- | --- | --- | --- | --- | --- | --- |
| **Moderator ^b^** | **OME/HL group** | **Interaction coefficient [95% CI]^c^** | **P-value** | **N** | **Interaction coefficient [95% CI]^c^** | **P-value** | **N** |
| HOME score 18 months | Unaffected | Reference | Reference | 789 | Reference | Reference | 629 |
|  | Mild/moderate | 0.16 [-1.83, 2.15] | 0.873 |  | -0.69 [-2.86, 1.47] | 0.529 |  |
|  | Severe | 3.87 [1.01, 6.73] | 0.008 |  | 2.18 [-0.99, 5.36] | 0.177 |  |
|  | **P for trend** | <0.001 |  |  | **P for trend** | 0.0817 |  |
| HOME score 30 months | Unaffected | Reference | Reference | 779 | Reference | Reference | 629 |
|  | Mild/moderate | 1.72 [-0.59, 4.04] | 0.144 |  | 1.63 [-0.83, 4.09] | 0.194 |  |
|  | Severe | 4.30 [1.39, 7.20] | 0.004 |  | 4.16 [0.66, 7.65] | 0.020 |  |
|  | **P for trend** | <0.001 |  |  | **P for trend** | 0.030 |  |
| HOME score 42 months | Unaffected | Reference | Reference | 774 | Reference | Reference | 629 |
|  | Mild/moderate | 0.98 [-1.08, 3.06] | 0.351 |  | 1.10 [-1.10, 3.30] | 0.327 |  |
|  | Severe | 2.81 [0.03, 5.59] | 0.047 |  | 2.98 [-0.20, 6.16] | 0.066 |  |
|  | **P for trend** | <0.001 |  |  | **P for trend** | 0.0428 |  |
