# Supplementary material for: Glue Ear, Hearing Loss and IQ: An Association Moderated by the Child’s Home Environment
Source: PLoS One. 2014 Feb 3;9(2):e87021. doi: 10.1371/journal.pone.0087021 (PMC3911938; doi:10.1371/journal.pone.0087021)
Supplement: Table S10 — Interactions between moderators and OME/HL (categorical variable) on performance IQ at age 8 years. a Adjusted for maternal education level, housing tenure, parental social class, maternal age, parity, smoking during 1st 3 months of pregnancy, smoking last 2 weeks of pregnancy, birthweight, gestational age, sex of child, HOME and parenting scores. b Moderators included if there was evidence of a significant interaction. c Coefficient of OME/HL and moderator interaction. The interaction effects reflect the change in the OME/HL effect compared to the reference level for not smoking in pregnancy (no smoking) or for a one unit change in the HOME score. Since the OME/HL effect is negative, positive interactions reflect an ameliorating effect. (DOCX) [file pone.0087021.s012.docx]

|  |  | **Unadjusted model** | | | **Fully adjusted model**^a^ | | |
| --- | --- | --- | --- | --- | --- | --- | --- |
| **Moderator^b^** | **OME/HL group** | **Interaction coefficient [95% CI]**^c^ | **P-value** | **N** | **Interaction coefficient [95% CI]**^c^ | **P-value** | **N** |
| HOME score 6 months | Unaffected | Reference | Reference | 800 | Reference | Reference | 631 |
|  | Mild/moderate | 0.79 [-0.86, 2.45] | 0.347 |  | 0.81 [-0.95, 2.58] | 0.369 |  |
|  | Severe | 2.81 [0.38, 5.23] | 0.023 |  | 2.09 [-0.48, 4.68] | 0.112 |  |
|  | **P for trend** | <0.001 |  |  | **P for trend** | 0.0743 |  |
| HOME score 18 months | Unaffected | Reference | Reference | 791 | Reference | Reference | 631 |
|  | Mild/moderate | 0.41 [-1.62, 2.45] | 0.688 |  | 0.87 [-1.39, 3.15] | 0.448 |  |
|  | Severe | 3.49 [0.57, 6.41] | 0.019 |  | 3.14 [-0.17, 6.45] | 0.063 |  |
|  | **P for trend** | <0.001 |  |  | **P for trend** | 0.1497 |  |
| HOME score 30 months | Unaffected | Reference | Reference | 781 | Reference | Reference | 631 |
|  | Mild/moderate | 2.30 [-0.08, 4.69] | 0.058 |  | 2.16 [-0.40, 4.74] | 0.099 |  |
|  | Severe | 4.82 [1.83, 7.80] | 0.002 |  | 4.71 [1.07, 8.35] | 0.011 |  |
|  | **P for trend** | 0.0044 |  |  | 0.0514 |  |  |
| HOME score 42 months | Unaffected | Reference | Reference | 776 | Reference | Reference | 631 |
|  | Mild/moderate | 2.10 [-0.01, 4.22] | 0.052 |  | 1.18 [-1.13, 3.49] | 0.317 |  |
|  | Severe | 3.75 [0.92, 6.58] | 0.009 |  | 2.58 [-0.72, 5.90] | 0.126 |  |
|  | **P for trend** | <0.001 |  |  | 0.0191 |  |  |
| Smoking last 2 weeks preg | Unaffected | Reference | Reference | 803 | Reference | Reference | 631 |
|  | Mild/moderate | -11.86 [-21.08, -2.65] | 0.012 |  | -12.78 [-23.03, -2.53] | 0.015 |  |
|  | Severe | -12.88 [-24.91, -0.86] | 0.036 |  | -15.99 [-30.16, -1.82] | 0.027 |  |
|  | **P for trend** | 0.010 |  |  | 0.047 |  |  |
