# Supplementary material for: Glue Ear, Hearing Loss and IQ: An Association Moderated by the Child’s Home Environment
Source: PLoS One. 2014 Feb 3;9(2):e87021. doi: 10.1371/journal.pone.0087021 (PMC3911938; doi:10.1371/journal.pone.0087021)
Supplement: Table S11 — Association between OME/HL score (continuous) and verbal IQ at age 4 and 8. a A negative coefficient indicates that as the OME/HL severity score increases, IQ decreases. b Adjusted for maternal education level, housing tenure, parental social class, maternal age, parity, smoking during 1st 3 months of pregnancy, smoking last 2 weeks of pregnancy, birthweight, gestational age, sex of child. c Adjusted for maternal education level, housing tenure, parental social class, maternal age, parity, smoking during 1st 3 months of pregnancy, smoking last 2 weeks of pregnancy, birthweight, gestational age, sex of child, HOME and parenting scores. (DOCX) [file pone.0087021.s013.docx]

|  | **Unadjusted model^a^** | | | **Adjusted model^b^** | | | **Adjusted model^c^** | | |
| --- | --- | --- | --- | --- | --- | --- | --- | --- | --- |
|  | **Coefficient [95% CI]** | **N** | **P-value** | **Coefficient [95% CI]** | **N** | **P-value** | **Coefficient [95% CI]** | **N** | **P-value** |
| **OME/HL score (up to age 4) and IQ at age 4** | -0.482 [-0.67, -0.28] | 971 | <0.001 | -0.473 [-0.66, -0.28] | 869 | < 0.001 | -0.39 [-0.60, -0.19] | 729 | <0.001 |
| **OME/HL score (up to age 5) and IQ at age 8** | -0.135 [-0.37, 0.10] | 810 | 0.274 | -0.130 [-0.37, 0.11] | 730 | 0.289 | -0.139 [-0.40, 0.12] | 629 | 0.292 |
