# Supplementary material for: Glue Ear, Hearing Loss and IQ: An Association Moderated by the Child’s Home Environment
Source: PLoS One. 2014 Feb 3;9(2):e87021. doi: 10.1371/journal.pone.0087021 (PMC3911938; doi:10.1371/journal.pone.0087021)
Supplement: Table S12 — Association between OME/HL score (continuous) and performance IQ at age 4 and 8. a A negative coefficient indicates that as the OME/HL severity score increases, IQ decreases. b Adjusted for maternal education level, housing tenure, parental social class, maternal age, parity, smoking during 1st 3 months of pregnancy, smoking last 2 weeks of pregnancy, birthweight, gestational age, sex of child. c Adjusted for maternal education level, housing tenure, parental social class, maternal age, parity, smoking during 1st 3 months of pregnancy, smoking last 2 weeks of pregnancy, birthweight, gestational age, sex of child, HOME and parenting scores. (DOCX) [file pone.0087021.s014.docx]

|  | **Unadjusted model^a^** | | | **Adjusted model^b^** | | | **Adjusted model^c^** | | |
| --- | --- | --- | --- | --- | --- | --- | --- | --- | --- |
|  | **Coefficient [95% CI]** | **N** | **P-value** | **Coefficient [95% CI]** | **N** | **P-value** | **Coefficient [95% CI]** | **N** | **P-value** |
| **OME/HL score (up to age 4) and IQ at age 4** | -0.362 [-0.57, -0.14] | 876 | 0.001 | -0.365 [-0.58, -0.14] | 870 | 0.001 | -0.27 [0.50, -0.04] | 730 | 0.020 |
| **OME/HL score (up to age 5) and IQ at age 8** | -0.170 [-0.41, 0.07] | 812 | 0.173 | -0.115 [-0.37, 0.14] | 732 | 0.379 | -0.097 [-0.36, 0.17] | 631 | 0.481 |
