# Supplementary material for: Glue Ear, Hearing Loss and IQ: An Association Moderated by the Child’s Home Environment
Source: PLoS One. 2014 Feb 3;9(2):e87021. doi: 10.1371/journal.pone.0087021 (PMC3911938; doi:10.1371/journal.pone.0087021)
Supplement: Table S13 — Differences in mean verbal IQ score at age 4 according to separate OME group and hearing loss variables (up to 4 years). a Hearing loss categorised as WRT >35 dBA. b Fully adjusted for all confounders and HOME/parenting scores. (DOCX) [file pone.0087021.s015.docx]

|  | **Unadjusted model** | | | **Adjusted model^b^** | | |
| --- | --- | --- | --- | --- | --- | --- |
| **OME group** | **Coefficient [95% CI]** | **N** | **P value** | **Coefficient [95% CI]** | **N** | **P value** |
| Unaffected | Reference | 205 |  | Reference | 131 |  |
| Intermediate | -1.63 [-3.70, 0.43] | 667 | 0.122 | -1.97 [-4.22, 0.27] | 426 | 0.085 |
| Highest 10% score | -6.40 [-9.57, -3.22] | 99 | <0.001 | -4.31 [-8.44, -0.17] | 54 | 0.041 |
| **HL at age 2 ½** |  |  |  |  |  |  |
| Normal |  |  |  | Reference | 477 |  |
| Hearing loss^a^ |  |  |  | -2.90 [-5.29, -0.50] | 134 | 0.018 |
| **HL at age 3 ½** |  |  |  |  |  |  |
| Normal |  |  |  | Reference | 558 |  |
| Hearing loss^a^ |  |  |  | 0.44 [-4.00, 3.11] | 53 | 0.806 |
