# Supplementary material for: Glue Ear, Hearing Loss and IQ: An Association Moderated by the Child’s Home Environment
Source: PLoS One. 2014 Feb 3;9(2):e87021. doi: 10.1371/journal.pone.0087021 (PMC3911938; doi:10.1371/journal.pone.0087021)
Supplement: Table S14 — Differences in mean performance IQ score at age 4 according to separate OME group and hearing loss variables (up to 4 years). a Hearing loss categorised as WRT >35 dBA. b Fully adjusted for all confounders and HOME/parenting scores. (DOCX) [file pone.0087021.s016.docx]

|  | **Unadjusted model** | | | **Adjusted model^b^** | | |
| --- | --- | --- | --- | --- | --- | --- |
| **OME group** | **Coefficient [95% CI]** | **N** | **P value** | **Coefficient [95% CI]** | **N** | **P value** |
| Unaffected | Reference | 206 |  | Reference | 131 |  |
| Intermediate | -0.30 [-2.56, 1.95] | 669 | 0.791 | 0.14 [-2.41, 2.71] | 426 | 0.910 |
| Highest 10% score | -5.24 [-8.70, -1.77] | 99 | 0.003 | -1.72 [-6.45, 2.99] | 54 | 0.473 |
| **HL at age 2 ½** |  |  |  |  |  |  |
| Normal |  |  |  | Reference | 477 |  |
| Hearing loss^a^ |  |  |  | -3.14 [-5.88, -0.41] | 134 | 0.024 |
| **HL at age 3 ½** |  |  |  |  |  |  |
| Normal |  |  |  | Reference | 558 |  |
| Hearing loss^a^ |  |  |  | -0.59 [-4.65, 3.46] | 53 | 0.774 |
